# New concepts for building vocabulary for cell image ontologies

**DOI:** 10.1186/1471-2105-12-487

**Published:** 2011-12-21

**Authors:** Anne L Plant, John T Elliott, Talapady N Bhat

**Affiliations:** 1 Biochemical Science Division, National Institute of Standards and Technology Gaithersburg MD 20899, USA

## Abstract

**Background:**

There are significant challenges associated with the building of ontologies for cell biology experiments including the large numbers of terms and their synonyms. These challenges make it difficult to simultaneously query data from multiple experiments or ontologies. If vocabulary terms were consistently used and reused across and within ontologies, queries would be possible through shared terms. One approach to achieving this is to strictly control the terms used in ontologies in the form of a pre-defined schema, but this approach limits the individual researcher's ability to create new terms when needed to describe new experiments.

**Results:**

Here, we propose the use of a limited number of highly reusable common root terms, and rules for an experimentalist to locally expand terms by adding more specific terms under more general root terms to form specific new vocabulary hierarchies that can be used to build ontologies. We illustrate the application of the method to build vocabularies and a prototype database for cell images that uses a visual data-tree of terms to facilitate sophisticated queries based on a experimental parameters. We demonstrate how the terminology might be extended by adding new vocabulary terms into the hierarchy of terms in an evolving process. In this approach, image data and metadata are handled separately, so we also describe a robust file-naming scheme to unambiguously identify image and other files associated with each metadata value. The prototype database http://sbd.nist.gov/ consists of more than 2000 images of cells and benchmark materials, and 163 metadata terms that describe experimental details, including many details about cell culture and handling. Image files of interest can be retrieved, and their data can be compared, by choosing one or more relevant metadata values as search terms. Metadata values for any dataset can be compared with corresponding values of another dataset through logical operations.

**Conclusions:**

Organizing metadata for cell imaging experiments under a framework of rules that include highly reused root terms will facilitate the addition of new terms into a vocabulary hierarchy and encourage the reuse of terms. These vocabulary hierarchies can be converted into XML schema or RDF graphs for displaying and querying, but this is not necessary for using it to annotate cell images. Vocabulary data trees from multiple experiments or laboratories can be aligned at the root terms to facilitate query development. This approach of developing vocabularies is compatible with the major advances in database technology and could be used for building the Semantic Web.

## Background

Cell images are a mainstay of cell biology data because of the vast amount of information that they can contain. In addition to information on the explicit analyte of interest, images contain information such as spatial and intensity relations that provide insight to the practitioner, even when the information is not explicitly extracted for formal analysis. With the advent of modern automated instrumentation, vast amounts of image data are being collected, and this huge volume of data poses a significant challenge to thorough analysis [1]; [2]; [3]; [4]. Because it is likely that there is more information embedded in cell image data than is usually being extracted, it is of great interest in individual labs to be able to locate stored image data easily for additional analysis and for comparison with other image data [5]; [6]; [7]; [8]. There is also interest in being able to share data between laboratories [9]; [10], allowing for independent analyses, validation of results, integration of data that explore different parameter space, and to combine results from different kinds of measurements [11] to understand cellular behavior. Many fundamental questions in biology and medicine will likely not be solved without better integration of data from different sources [12]. There are many challenges associated with data sharing [9], and many of these have been acknowledged for a decade but never completely solved [13].

A particular challenge associated with sharing biological research data is that there are many experimental parameters and descriptive terms associated with studies involving cells. Documenting these experimental details is critical for the effective exchange and use of primary data. A number of metadata activities have arisen in an attempt to specify some experimental conditions and terminology for cell-based assays, including Minimum Information About a Cell Assay [14]; Minimum Information about Flow Cytometry [15] and Minimum Information About T cell Assays [16]; [17]. Other related ontologies include the Cell Ontology (CL) which focuses on controlled vocabulary for cell types [18], The Microarray Gene Expression Data group (MGED) [19] which has developed a list of terms to describe cell-based experiments, The Open Microscopy Environment (OME) consortium [20]; [5] which has developed terms that describe microscope equipment, and a systems biology group [21] that has developed an ontology for describing high content imaging experiments. For the most part, these activities have focused on schema for acquiring experimental metadata.

Most metadata activities such as those mentioned above are established to serve a specific experimental or application niche, and to serve a relatively narrow community. These efforts have produced different ontologies with different terms, and with different organizational structures and relationships between terms. As a result, it is largely impossible to query across databases that cover different biology and biomedical domains [22]; [23]; [12]. The Semantic Web is predicated on the idea that unique insight will be gained from querying different types of data that reside in different databases. An example would be to combine information in clinical data in the Cancer Biomedical Informatics Grid (CaBIG) with cell biology data. The Cell Ontology (CL) uses the term 'cell' as a node term, and classifies cells in organisms according to organsm type, class, etc. The Semantic Nomenclature in Medicine (SNOMED) [24] on which CaBIG is organized does not use the term 'cell', although 'cell' is a component of many terms. Because they do not share 'cell' as a node, these ontologies do not easily intersect using the term 'cell'. A number of activities have been undertaken to address impediments like this. The Semantic Web aims to achieve communication between databases through the use of the Resource Description Framework language and other technologies [12]. Efforts to standardize terms for biological experiments have been undertaken by the Open Biological and Biomedical Ontologies (OBO) [25], and the mapping of schema and defining organizational structure such as have been embodied in BioMART [26]. BioMart provides software that allows users to map pre-existing databases to one another by creating a shared ontology, thereby allowing queries across ontologies by establishing terminology nodes that form intersection points across federated ontologies.

In this report, we begin to explore a different way of developing shared terms for organizing vocabularies (i.e., 'roots'), and vocabulary of high granularity that can be used to search cell imaging data. Since very few annotated cell image databases currently exist (a notable exception is the American Society of Cell Biology's Cell Image Library) [27], there is an opportunity to experiment with a new approach to the development of terminology for this challenging application. We examine the possibility that a sufficiently general structure for terminology organization can allow a natural expansion of terms, and provide intersection points with many different kinds of biological data. In this report, we build on the ideals of the Semantic Web to suggest rules by which terminology that describes cell biology experiments can be chosen and organized that facilitate evolution of ontologies and enable semantic queries of multiple datasets simultaneously. With this approach, sharing root terms such as 'cell' allows ontologies to be easily combined. We apply this approach in a prototype imaging database that aims to capture and display metadata that describe experimental details at a high level of granularity. We describe how local control of terms can be achieved while allowing the sharing of terms across distributed datasets, and how different queries can be developed on demand from the same set of metadata terms.

## Results

### Building Vocabulary Hierarchies Using Root Terms

**Figure 1:**
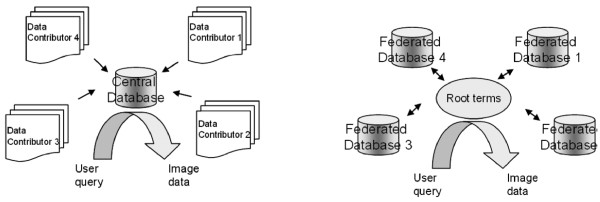
**Common root terms allow intersection between distributed databases**. Instead of all data being supplied to a central database before it can be queried (as depicted on the left), decentralized data can be accessed for querying provided that they share common root terms.

A common approach to ontology development is to codify specific vocabulary terms and organize them into schema. While this approach has advantages, the disadvantages are that schema cannot easily evolve to accommodate new terms and relationships [28]. An alternative to a predefined ontology is to develop a strategy or rules for spontaneous addition of terms under established 'root' terms as needed by federated partners. Because of the size of image files, it is advisable to consider a system of federated sites for image data storage. Root terms provide points of intersection between distributed databases (Figure 1). In this approach, we represent the hierarchy of terms as nested folders, which does not require naming the relationship between terms. Examples of rule-based hierarchies include cheminformatics software such as Chem-BLAST [29], ChEBI [30], and InChI [31]. In Chem-BLAST for example, terms are established using automated procedures based on certain rules that connect atoms to form a molecule [29]. Some of these principles for design of organizational rules and addition of new terms can be adopted to guide the development and addition of terms that describe experimental parameters in cell imaging data.

An essential rule is the use of root terms which serve as the basis for organizing more specific vocabulary terms under them. Root terms are commonly used in existing ontologies (eg., Gene Ontology (GO) [32] and SNOMED [24], but to be useful for bridging different databases, root terms need to be sufficiently general. For a cell imaging vocabulary, initial root terms will have to be agreed on by the community in a 'top-down' fashion as being terms that are highly reused within and across different databases. From then on, a 'bottom-up' approach can be envisioned, where a developer with a new use case will use the common root terms when possible under which to add new descriptive terms. These new terms may be terms that are likely to be reused by others, but the developer may also add terms that are very specific to his specialized use case, which are unlikely to be reused by others. In this way, a vocabulary structure specific to a use case can be developed while maintaining links to other ontologies through common terms. Using concepts of Semantic Web, the organization of terms is based on a use case; in other words, terms are chosen because they might be used to pose a question about the details of the experiment. The terms are organized into a predictable data tree structure, which could be used to build an RDF structure or a formal ontology by the addition of relationships and restrictions.

**Figure 2:**
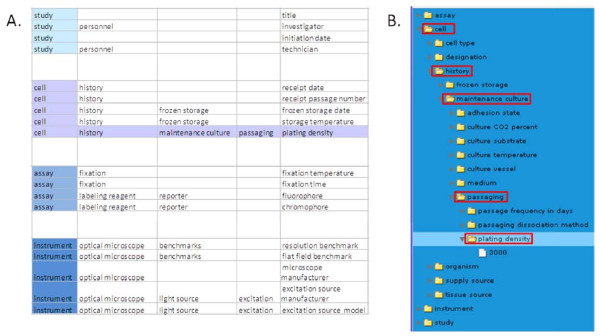
**Organization of terms**. Some examples of the use of a spread sheet to organize terms within broad categories (*study, cell, assay, instrument*) and concatenating them into logical phrases (i.e. cell:history:maintenance_culture:passaging:plating_density) (A). The user develops the query through these terms through a series of folders (B).

Considering the need for harmonization of vocabulary across multiple databases, we have chosen for our initial effort at selecting root terms the words *study, assay, cell*, and *instrument*. These are short terms with meanings that represent general and fundamental components of the experimental information which are used frequently in many existing biology ontologies. Under the root term *study *are values for details similar to those in IsaTAB [33], such as where and when the study has taken place, the names of the principle investigator and the people who performed the experiments, and the title of the study. Under the root term *assay *are found terms that describe assay details, such as how the series was collected, the seeding density of cells, post-experiment processing such as fixation, the assay target, and the reagents used. Under *cell *are terms that describe the source, identification, and routine handling, passaging and culture conditions for the cells that were ultimately used in the study. *Instrument *contains terms that identify the microscope and components, as well as materials used to benchmark instrument performance. The data tree presents a folder for the root term *cell*, which can be expanded to display other dependent folders such as *history*, which can then be expanded to expose terms such as *maintenance culture*. New vocabulary terms can be added at any and all levels of the hierarchy as necessary based on their relationship to existing terms, and root terms may be placed at any level of an ontology as needed. Figure 2A shows in a spreadsheet form examples of how root terms can be concatenated with more specific terms into logical phrases that describe experimental data. The complete list of metadata terms describing the cell experiments is provided in Additional File 1: Table of current metadata terms.

Some suggested rules for maintaining order in the creation of terms are articulated in Table 1. The following questions should drive the selection of terms: 1) What are common descriptive terms that are associated with many of the experiments in our database? 2) What are the most likely questions a user would like to ask (ie., use-cases) to understand these experiments? 3) What are the most reusable, adequately granular, short but unambiguous terms that can be used to describe the experiments in the context of the use-case queries? 4) Have we chosen reusable terms that are likely to appear in other ontologies in fields related to ours? The informal relationships between the terms are based on the logic a user may apply to find answers to his questions. We assume that usually a user starts of with a very general concept and then converges to concepts that form precise questions.

**Table 1:** Some 'rules' for adding vocabulary terms and metadata tokens to a vocabulary hierarchy.

Ideally, new terms will...
... be added only if they are required for a semantic query.
... be placed within root categories under which they logically belong.
... create new root categories only when required.
... be short words or phrases to facilitate sharing between databases.
... be substantially different from existing terms (e.g., cell line = cell_line = cellline).
... consider terminology from existing ontologies or vocabulary structures.
... be subject to change if another highly reused synonym is identified.

Visual query tools can present these terms as a data tree of expandable folders and subfolders. Arranging vocabulary into expandable folders based on root terms and nested folders of increasing specificity allows the user to logically identify metadata that suit their query needs. This concept is shown in Figure 2B. This approach allows developers the flexibility in creating terms and organizing them, and visualization of terms makes searching unambiguous and intuitive for the user. The data tree explicitly shows the available terms from which the user can choose terms on which to base their query. Different terms can be selected to construct different queries. Having the user select visualized terms is more efficient than requiring the user to provide search terms because only those terms that are represented in the database can be selected, and the user is spared the frustration of searching on terms that don't exist. In addition, by explicitly showing the available terms, insignificant differences in vocabulary, such as capital letters, dashes, and 'stop words ' such as 'of' and '()' can be ignored by a user. In this way, visualization enables the harmonization of different vocabularies.

Reuse of terms by developers is critical to the success of this approach because they are the basis of alignment of vocabulary hierarchies. For example, if a database has two sets of vocabulary hierarchies, one set that uses the term 'instrument' and others uses its synonymous term 'apparatus' to describe the same concept, the alignment technique may fail. This may result in a user overlooking relevant data or the need to open up multiple folders instead of one to view all the relevant data.

### A Layered Approach for Metadata and Image Data

Microscopy image data frequently include metadata in the form of image file header information. An alternative approach is to handle the two different kinds of data, metadata and image data, separately in order to satisfy their specific requirements. The image data (or data from any experimental instrument) must be immutable. Any processed image data or analytical results such as masks derived from image data, for example, would constitute a new file. Because image data files can be large and numerous, this presents storage and maintenance challenges which can be reduced if they can reside as flat files at geographically dispersed locations throughout the federation. On the other hand, it is desirable that the metadata terms can be updated and expanded, and the vocabulary hierarchies can evolve and be shared among users. To facilitate this, the metadata terms are organized in a data table or content graph that is handled separately from the image data. Data tables allow flexible expansion and organization of data, and rapid searching, using semantic Web concepts or standard SQL queries through the use of modern database indexing technology. Data tables can also be more easily shared among experimentalists and sites than image data can.

Using the broad guidance provided by root terms, independent developers and experimentalists can add new metadata terms as needed simply by appending new terms to their local data table. Vocabulary hierarchies can thus evolve as these new terms are added. Because of the ease of sharing data tables, the various data tables from different laboratories and sites can be combined at any time. We envision a complete vocabulary structure that would be comprised of a combination of terms from different vocabulary hierarchies that were developed independently at different federated sites using the same root term structure. All terms would be combined, and related terms would be organized under common nodes. Terms would be presented visually in an expandable folder structure, from which desired terms would be chosen as the basis of queries on demand. Through the connection of metadata terms with image data files, data from all sites would be accessible through a single query of the combined vocabulary structures. It is important to note that different laboratories may use different terms to describe similar experimental parameters. In fact, individual sites may have an abbreviated set of local metadata terms that are sufficient for their local needs. However, if all sites use shared root terms, the terms can be organized within common nodes, and it will be possible to intersect between the different data.

An example of how the metadata can be spontaneously expanded and evolved is described in Additional File 2: Adding new metadata terms to the existing ontology. The current prototype cell imaging database is described by metadata for many experimental parameters, but so far the database does not include time-dependent image data. In our vocabulary hierarchy, metadata describing time-dependent experimental conditions can be best appended to the existing metadata under the root term *assay*. A new folder (or node) such as *time lapse *can be added under the *collection basis *folder which is within the *image series details *folder. Within the *time lapse *folder can be added terms such as *total time, time interval*, etc. If metadata terms for new experiments are chosen thoughtfully, i.e., if established root terms are used whenever possible, new terms are at an appropriate level of granularity, and new root terms are created as needed to group related terms, the new added terms are more likely to be reused by other experimentalists.

### A File-naming Rule

In order to implement a layered approach to storing image data and metadata separately, it is essential to have a robust file naming scheme to unambiguously associate appropriate metadata with every image in the database. We use a file naming scheme based on Ontological Unique Resource Identifiers (OURI) [34]; [35]. OURIs can map to Unique Resource Identifiers (URIs) in the same way that Unique Resource Names (URNs) can be mapped as Persistent Uniform Resource Locators (PURLs). Metadata terms are linked to image and protocol data filenames with three reference data tables that are shown in Additional File 3: Data tables to link metadata to image and protocol data filenames.

**Figure 3:**
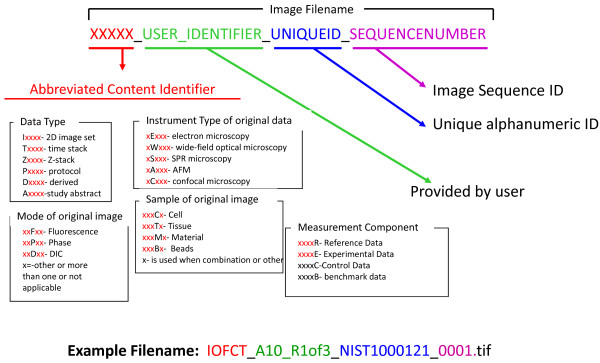
**An Ontological Unique Resource Identifier (OURI) Filenaming Scheme**. This is the file naming convention used for the current prototype database. This naming scheme is also used for the free-text files that are used to describe the protocols and the study abstracts of the image series.

The file naming scheme for the OURIs is composed of four parts: an abbreviated content identifier, a user-defined region, a unique ID, and an image series number, as is illustrated in Figure 3. In our current implementation, the abbreviated content identifier provides one-letter codes to describe the type of file. In our prototype database, we consider image data files, free-text protocol files, reference or benchmark data files, and files containing derived data. Other kinds of information about the file content can be codified as seen in Figure 3. We accommodate the common use of descriptive text in biological research with the user-defined region. This area could also be used by the experimentalist to provide other information, such as information about original folder structure of the image files, to facilitate file organization. Also, the small amount of text information in the abbreviated content identifier and the user-defined region provide to the user an intuitive check that the data returned are consistent with the data expected. The combination of a unique ID and an image series number ensures that no two files can have the same name. A federated site would have local control over this unique ID, which could also allow identification of the site.

### The Prototype Database

**Figure 4:**
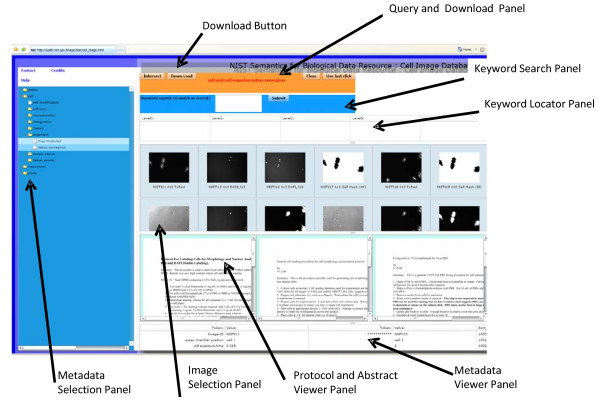
**Main Query Page of the Prototype Database**. See Additional File 4 for a complete description of the website.

We present a prototype cell image database in order to illustrate some of the concepts described above. This prototype database allows queries to be developed on demand for probing experimental differences at high granularity between nominally identical datasets. The data in the prototype database http://sbd.nist.gov/image/cell_image.html are NIST Standard Reference Data #165. The images in the prototype database were collected to facilitate a comparative study of image segmentation algorithms [36]. The database contains image data from replicate wells for two cell types that were collected under 5 different imaging conditions. Images of spatial, resolution, and intensity benchmarks are also included. The database also contains masks corresponding to each cell object that were determined by manual segmentation. The mask data are found under in the metadata tree under *assay*:*datatype*:*derived*. It is important to note that we did not include metadata fields that would allow long free-form text descriptions of details as metadata values. Instead these free-form descriptions are placed in separate files that are displayed when an image series is selected (see Figure 4, Protocol and Abstract Viewer Panel). More information about the study that generated these data are in the 'help' section of the website.

Figure 4 shows an example of a main query webpage from the prototype database. The main panels of the webpage are described in the Additional File 4: The Prototype Database Screens.

## Discussion

Given the enormous number of parameters to consider in cell biology, achieving predictive understanding of complex biological systems will require that experimentalists and modelers have access to more well-characterized data. The ability to search multiple datasets simultaneously based on experimental details, or other criteria such as image features or the results of analysis, could greatly expand the usefulness of imaging data. Simultaneous semantic querying of multiple datasets could enable hypothesis testing, and could also help to identify the critical parameters that influence experimental outcomes. Being able to navigate multiple imaging datasets could allow systematic comparison of image handling and analysis procedures. Imaging data could be reanalyzed and mined for additional relationships and insight. The results of image data could be combined with data from other types of measurements such as gene expression analysis. Advanced concepts for identifying and organizing metadata terms are needed to achieve this inter-laboratory and inter-database data exchange.

With these goals in mind, we are experimenting with concepts that would enable the development of local imaging databases that can be searched simultaneously by semantic query on vocabulary terms that describe the various facets of the experiment or the experimental results. A critical requirement for performing queries across databases is shared vocabulary terms. Our prototype database contains a rather limited number of metadata terms, which are sufficient to describe the experiments represented by the current prototype imaging data. Additional terms to describe other types of imaging experiments can be added as necessary by any user to accommodate new datasets. We offer rules for adding new terms into a root-based hierarchical structure. The development of consensus root vocabulary terms is ongoing and will be an evolving process through community activity.

The selection of ideal root terms is not trivial, and we anticipate that this process will evolve by trial and error, and through the use of algorithms that will determine frequency of term usage. software that accumulates word usage in the biology field. Root terms should be sufficiently broad that they can serve as useful nodes for organizing dependent terms under them. If root terms are too broad, there will be too many terms under them for comfortable viewing. Terms that are too specific will not be shared by other databases.

A critical concept that makes this vision possible is the focus on short and highly reusable terms, vocabulary hierarchies and data tables instead of rigid schema and relationships to build and organize a specific ontology. Many of the minimum information efforts and other ontology development activities have developed schema as part of their implementation. While such an approach allows unambiguous presentation of data, and is necessary for metadata capture, schema can constrain terminology [9]; [25] to a list of terms that is accepted at that moment in time. The speed at which biological science is progressing, and the variety of experiments and data that practitioners would like to access, make such a rigid approach quite limiting [28]. Here, we suggest an approach for building vocabulary hierarchies based on root terms. Root terms provide a framework and a guide for addition of new terms and context in the vocabulary structure. Additions can be made spontaneously at a local level, as demanded by new experimental descriptions. In this way, vocabulary can be developed locally but still share terms with other vocabulary hierarchies and ontologies. Because the metadata layer is handled separately from the image data layer, metadata terms from all vocabulary structures and all federated sites can be combined and viewed within an aligned data-tree structure, and in this way, as any database expands the list of metadata terms, other users can see and reuse the new terms. Root terms and their context form the basis of semantic queries across the vocabulary hierarchies, allowing different databases with other vocabulary structures to be searched simultaneously.

Visualization of terms within a hierarchical data tree structure make selection of query terms unambiguous. This approach to metadata development and organization allows a natural evolution of vocabulary terms while maintaining harmonization across different metadata development efforts. This approach employs both 'top-down' and 'bottom-up' approaches to defining, reusing, and extending terminologies.

## Conclusion

The prototype database presented here contains a limited amount of imaging data, but allows us to explore many of the concepts for cell imaging databasing that would be compatible with an expanding and flexible vocabulary and a federated system of databases. Future work will involve testing these concepts in a federated environment.

## Methods^i^

### Cell image data

This dataset was collected as part of a study to examine the effects of imaging conditions and cell type on the accuracy of segmentation operations. Rat A10 vascular smooth muscle cells and mouse NIH3T3 fibroblasts were seeded on tissue culture polystyrene dishes at a density of 800 cells/cm^2 ^and 1200 cells/cm^2^, respectively. Three replicate wells were prepared for each cell type. The cells were fixed with formaldehyde and treated with Texas Red-C2-maleimide which labels cellular proteins containing sulfhydryl groups providing an excellent stain for the cell body, and DAPI, which labels the cell nuclei. Fifty fields in each well were imaged by automated microscopy with a 10 × objective using phase contrast and fluorescence from Texas Red and DAPI [35]. The dataset also includes Texas Red fluorescence images that were collected to allow assessment of the effect of exposure time and sub-optimal filter conditions. These conditions resulted in significant variation in the signal-to-noise ratio and spatial resolution in the different image sets. The complete dataset includes 750 Texas Red images for each cell type (50 fields per well, 5 imaging conditions, and 3 replicate wells), plus 150 corresponding fields of DAPI fluorescence and phase images for each cell type, and 100 images of cell object masks determined by manual segmentation for each cell type.

Additional images of spatial calibration reference materials (1 image), intensity benchmarks (4 images), and a resolution target (2 images) were used to benchmark the microscope imaging system to facilitate future comparisons of this image datasets with other datasets.

### The Prototype Database

Metadata for each image series are collected by the experimentalist in Excel spreadsheets, and protocol documents are provided as MS Word documents. The experimentalist provides image data in TIF format, named according to their chosen filename, and add the Abbreviated Content Identifier (described in Figure 3). All experimentalist-provided files are modified automatically by the addition of a unique ID using a Perl script which imposes a file naming convention to provide a unique name for each file (as described in Results).

The data tables are assembled in Oracle which associates appropriate metadata, protocols and image data file names with one another (see Additional File 3). The prototype database runs on a Sun MicroSystem server. The database is transferred to a MySQL database for public viewing. Queries are processed using Web services. For rapid visualization, image and protocol files are displayed as PNG files. Original data are stored as TIF (for image data) or MSWord documents (for text data). The entire dataset consists of approximately 2300 images with corresponding metadata and protocol files.

The url for the cell image database is http://sbd.nist.gov/.

## Authors' contributions

ALP: development of metadata terms, manuscript preparation; JTE: development of metadata terms, collection of images, organization of data, manuscript preparation; TNB: development of concepts, programming, manuscript preparation. All authors read and approved the final manuscript.

## Foot Note

^i ^Commercial products are identified in this article to adequately specify the experimental procedure. This does not imply recommendation or endorsement by the National Institute of Standards and Technology, nor does it imply that the materials or equipment identified are necessarily the best available for the purpose.

## Supplementary Material

Additional File 1 **The current metadata terms**. A complete list of the metadata terms that are used to describe the experimental conditions for the cell images in the prototype database. For the particular use case described, the terms are organized in the hierarchical structure shown and can be visualized in the form of nested expandable folders (e.g, study, personnel, etc). The left-hand columns contain the most general and reused terms that are the most likely terms for intersection with other databases. The token column contains the most specific terms, and the final column contains the metadata values. The metadata values shown in this table are examples of values that may be used to describe an experiment. We have attempted to use vocabulary where the definition of the term is obvious and unambiguous (i.e. human readable). A complete term would be the concatenation of the metadata token and preceding terms, e.g., study:personnel:investigator:John Elliott. There terms are not absolute. If another term is more reused and acceptable as a synonym for a specific concept, then the term in this database should be changed to maximize interoperability. Click here for file

Additional File 2 **Adding new metadata terms to the existing ontology**. New terms are added to describe time lapse data. The new lines of terms can be added to the end of the existing metadata list or data table shown in Additional File 1. The new terms (which are highlighted) are associated with appropriate existing root terms as shown. When the data tree is generated from the data table, terms are grouped into common nodes defined by the root terms. Click here for file

Additional File 3 **Data Tables used for Linkage between Metadata and Image and Protocol Files**. Data tables showing linkage between metadata terms and unique filenames for the image and protocol data. Three tables are generated during the upload of the metadata template file, the image file series and the protocol files. The file series name connects the tables. Click here for file

Additional File 4 **Description of the Prototype Database Screens**. The url for the database is http://sbd.nist.gov/image/cell_image.html. This file contains information about navigating the web page. Click here for file
