# Supplementary material for: New concepts for building vocabulary for cell image ontologies
Source: BMC Bioinformatics. 2011 Dec 21;12:487. doi: 10.1186/1471-2105-12-487 (PMC3293096; doi:10.1186/1471-2105-12-487)
Supplement: Additional File 1 — The current metadata terms. A complete list of the metadata terms that are used to describe the experimental conditions for the cell images in the prototype database. For the particular use case described, the terms are organized in the hierarchical structure shown and can be visualized in the form of nested expandable folders (e.g, study, personnel, etc). The left-hand columns contain the most general and reused terms that are the most likely terms for intersection with other databases. The token column contains the most specific terms, and the final column contains the metadata values. The metadata values shown in this table are examples of values that may be used to describe an experiment. We have attempted to use vocabulary where the definition of the term is obvious and unambiguous (i.e. human readable). A complete term would be the concatenation of the metadata token and preceding terms, e.g., study:personnel:investigator:John Elliott. There terms are not absolute. If another term is more reused and acceptable as a synonym for a specific concept, then the term in this database should be changed to maximize interoperability. [file 1471-2105-12-487-S1.RTF]

Additional File 1:  The current metadata terms.   A complete list of the metadata terms that are used to describe the experimental conditions for the cell images in the prototype database.   For the particular use case described, the terms are organized in the hierarchical structure shown and can be visualized in the form of nested expandable folders (e.g, study, personnel, etc).  The left-hand columns contain the most general and reused terms that are the most likely terms for intersection with other databases.  The token column contains the most specific terms, and the final column contains the metadata values.  The metadata values shown in this table are examples of values that may be used to describe an experiment.   We have attempted to use vocabulary where the definition of the term is obvious and unambiguous (i.e. human readable).  A complete term would be the concatenation of the metadata token and preceding terms, e.g., study:personnel:investigator:John Elliott.  These terms are not absolute.  If another term is more reused and acceptable as an alternative for a specific concept, then the term in this database should be changed to maximize interoperability.  This process is how the vocabulary would evolve for better harmonization.
Folder Level 0	Folder Level 1	Folder Level 2	Folder Level 3	Metadata Token	Metadata Values (example)	Definition of Token	
<NOTE-  "study" is used as a root folder term and holds variables that are related to who, when and where of the study and the name of the study>	
study				title	Morphology of NIH3T3 nuclei	uncontrolled text <100 characters. A constant for related replicate, control and benchmark image files from a single study.  This term describes the  title of the study.	
study	personnel			investigator	John Elliott	Designer of the study. A constant for identifying related series in a study	
study				initiation date	4/16/2008	Date that a study was initiated.  A constant for replicates, controls and benchmark samples	
study	personnel			technician	John Elliott	Laboratory person who collected this series of data	
study	personnel			institution	NIST	The institution  that is linked to the investigator	
<NOTE- "cell" is used as a root folder term and holds all the variables that are related to the cell line used in the study>	
cell				designation	NIH-3T3	Name of the cells being used in the study	
cell				supply source	ATCC	Vendor or location where the cells were obtained	
cell				organism	mus musculus	The genus species of the cells	
cell				tissue source	embryo	tissue source for cells	
cell				cell modification		Genetic or other modifications that have been introduced into the cell	
cell				cell type	fibroblast	The type of cell (endothelial, fibroblast, etc)	
cell	history			receipt date		The date the cell line was received in the laboratory	
cell	history			receipt passage number		The passage number of received cell stock	
							
cell	history	frozen storage		frozen storage date		The date the current culture was frozen	
cell	history			thaw date		The date the current culture was thawed	
cell	history	frozen storage		storage passage number		The passage number at which current culture was frozen	
cell	history	frozen storage		storage medium	95% culture medium+5% DMSO	Solution composition in which cells were frozen	
cell	history	frozen storage		storage temperature	-135	Temperature at which frozen cells were stored (deg C)	
cell	history	maintenance culture	passaging	plating density	3000	Density at which cells in the maintenance culture were plate for routine passage (cells/sq cm)	
cell	history	maintenance culture	passaging	passage frequency in days	3	The frequency at which cells in the maintenance culture were routinely passaged (eg, 2-3 d)	
cell	history	maintenance culture	passaging	passaging dissociation method	trypsin	Highly reused terms to describe the method used to dissociate the cells from the maintenance culture flask during passage (trypsin, EDTA, etc)	
cell	history	maintenance culture		adhesion state	adhered	Indicates if the cells in the maintenance culture are adhered or suspended	
cell	history	maintenance culture		culture substrate	tissue culture polystyrene	Highly reused terms describing the adhesion surface for routine passaging of the maintenance culture (TCPS, gelatin)	
cell	history	maintenance culture		culture vessel	T-25 flask	The name of the container used for routine passaging of the maintenance culture	
cell	history	maintenance culture		culture temperature	37	The temperature for incubation of the maintenance culture (deg C).	
cell	history	maintenance culture		culture CO2 percent	5	The fraction of CO2 in incubator for the maintenance culture (in percentage)	
cell	history	maintenance culture	medium	culture medium name	DMEM	The name of the culture media used for the maintenance culture	
							
cell	history	maintenance culture	medium	culture medium source	Mediatech	The vendor name for the culture media used to store the maintenance culture	
cell	history	maintenance culture	medium	culture medium catalog number		The catalog number for the culture media used to store the maintenance culture	
cell	history	maintenance culture	medium	culture medium antibiotic supplement	penicillin+streptomycin	The name of the antibiotic supplement used in the maintenance culture	
cell	history	maintenance culture	medium	culture medium amino acid supplement	non-essential amino acids+glutimax	The name of the amino acid supplements used in the culture media	
cell	history	maintenance culture	medium	culture medium hormone supplement		The name of the hormone used in the maintenance culture	
cell	history	maintenance culture	medium	culture medium serum type	fetal bovine	The type of the serum used in the maintenance culture media	
cell	history	maintenance culture	medium	culture medium serum percent	10	The percent of volume of serum in the maintenance culture media	
cell	history	maintenance culture	medium	culture medium serum source		The source of the serum used in the maintenance culture media	
cell	history	maintenance culture	medium	culture medium serum catalog number		The catalog number of the serum used in the maintenance culture media	
cell	history	maintenance culture	medium	culture medium serum lot number		The lot number  of the serum used in the maintenance culture media	
cell	characteristics			mean cell doubling time		The measured mean cell doubling time (in hours)	
cell	characteristics			mean cell volume		The measured mean cell volume (cubic microns)	
cell	characteristics			genetic identification		Genetic tag information that provides a genetic ID of the cells	
cell	characteristics			mean cell spread area		The mean spread area of the cell adhered to TCPS  	
<NOTE- "assay" is used as a root folder term and holds all the variables involved in the assay>	
assay	image series details			collection basis	fields	The basis of the images in the series.   For example, fields in a well, wells, time, microfluidic chambers, etc 	
assay	image series details			experiment type	cell morphology	Highly reused terms providing short description of the type of experiment  cell morphology, immunohistochemistry, timecourse, microfluidic,  	
assay	image series details			measurement component	experimental sample	Highly reused terms that provide a short description of how the image series plays a role in the measurement.  For example, positive control, negative control, spatial benchmark, intensity benchmark, experimental sample, derived data. 	
assay	image series details			image collection date	4/20/2008	The date the image series was collected	
assay				measurement endpoint	cell area	Highly reused words that describe the assay measurement that will be generated from the image series. 	
assay	cell handling	chamber 		assay chamber format	6-well plate	The type of chamber format that is being used for cell assay.	
assay	cell handling	chamber 		assay chamber temperature	37	The temperature of the incubator being used for the cell assay (deg C)	
assay	cell handling	chamber 		assay chamber substrate	tissue culture polystyrene	The material used to prepare the cell adhesion substrate of the assay chamber.	
assay	cell handling	chamber 		assay chamber position 	well 4	Highly reused words describing the position in the assay chamber ( plate, well position)	
assay	extracellular matrix			extracellular matrix molecular structure		Highly reused words that describe the structure of the proteins that are used to form the extracellular matrix.monomer, fibril	
assay	extracellular matrix			extracellular matrix protein		Highly reused words that describe the principle protein component of the extracellular matrix	
assay	extracellular matrix			extracellular matrix source		The source of the extracellular matrix protein.	
assay	extracellular matrix			extracellular matrix lot number		from supplier, or date from lab	
assay	extracellular matrix			extracellular matrix solution concentration		During deposition in mg/mL	
assay	extracellular matrix			extracellular matrix deposition solvent		Highly reused terms describing the carrier solution for protein during deposition	
assay	extracellular matrix			extracellular matrix incubation time		Hours of deposition	
assay	extracellular matrix			extracellular matrix incubation temperature		Temperature of incubation during deposition of matrix (in deg C)	
assay	extracellular matrix			extracellular matrix patterning		Highly reused terms describing the patterning of the extracellular matrix	
assay	extracellular matrix			extracellular matrix treatment		Highly reused terms describing additional treatment of the extracellular matrix.	
assay	extracellular matrix			extracellular matrix other components		Highly reused terms for protein, nonprotein additives	
assay	cell handling			assay passage number		Passage number of when cells are used in experiment	
assay	cell handling			assay seeding density	1000	seeding density for experiment; cells/cm2	
assay	cell handling			preseeding dissociation method	trypsin	Highly reused name describing the dissociation method used immediately prior to seeding for experiment	
assay	cell handling	medium		assay medium name	DMEM	conditions used for experiment	
assay	cell handling	medium		assay medium source	Mediatech	conditions used for experiment	
assay	cell handling	medium		assay medium catalog number		conditions used for experiment	
assay	cell handling	medium		assay medium antibiotic supplement	penicillin+streptomycin	conditions used for experiment	
assay	cell handling	medium		assay medium amino acid supplement	non-essential amino acids+glutimax	conditions used for experiment	
assay	cell handling	medium		assay medium hormone supplement		conditions used for experiment	
assay	cell handling	medium		assay medium serum type	fetal bovine	conditions used for experiment	
assay	cell handling	medium		assay medium serum percent	10	conditions used for experiment	
assay	cell handling	medium		assay medium serum source		conditions used for experiment	
assay	cell handling	medium		assay medium serum catalog number		conditions used for experiment	
assay	cell handling	medium		assay medium serum lot number		conditions used for experiment	
assay	cell handling	chamber 		assay chamber O2 percent		conditions used for experiment	
assay	cell handling	chamber 		assay chamber CO2 percent	5	conditions used for experiment	
assay	treatment			treatment1 parameter name		Highly reused terms for a chemical or physical treatment in the assay, even if this sample is a negative control	
assay	treatment			treatment1 concentration		Concentration of treatment 1	
assay	treatment			time of treatment1 after seeding		hours after seeding cells that treatment was added	
assay	treatment			time treatment1 removed		hours that treatment is in contact w cells	
assay	treatment			treatment2 parameter name		Highly reused terms for chemical or physical treatment in the assay, even if this sample is a negative control	
assay	treatment			treatment2 concentration		Treatment 2 concentration	
assay	treatment			time of treatment2 after seeding		hours after seeding cells that treatment was added	
assay	treatment			time treatment2 removed		hours that treatment is in contact w cells	
assay	fixation			time of fixation after seeding	20	hours after seeding that cells were treated with fixative	
assay	fixation			fixative	paraformaldehyde	Highly reused terms describing the fixative used in the assay	
assay	fixation			fixative concentration	1	Percent by volume	
assay	fixation			fixative concentration units	% volume fraction	Highly reused terms describing the units for fixation.	
assay	fixation			fixative buffer	phosphate buffered saline	Highly reused terms that describe the fixative buffer	
assay	fixation			fixation temperature	25	degrees C	
assay	fixation			fixation time	3	Hours fixative is in contact w sample	
assay	labeling reagent	reporter		fluorophore	DAPI	Highly reused term for the fluorescent labeling reagent	
assay	labeling reagent	reporter		chromophore		Highly reused term for describing the Non-fluorescent chromophore stain	
assay	labeling reagent	chemical label		chemical labeling reagent name	DAPI	Agent that perfoms chemical modification 	
assay	labeling reagent	chemical label		chemical labeling reagent concentration	3.6	when labeling reaction is part of the assay; in mg/mL	
assay	labeling reagent	chemical label		chemical labeling reagent incubation time	2	in hours	
assay	labeling reagent	affinity	antibody	primary antibody		The name or designation of the primary antibody	
assay	labeling reagent	affinity	antibody	primary antibody species		primary antibody species	
assay	labeling reagent	affinity	antibody	primary antibody source		primary antibody source	
assay	labeling reagent	affinity	antibody	primary antibody lot number		primary antibody lot number	
assay	labeling reagent	affinity	antibody	primary antibody type		primary antibody type	
assay	labeling reagent	affinity	antibody	primary antibody working concentration		primary antibody working concentration	
assay	labeling reagent	affinity	antibody	primary antibody incubation time		primary antibody incubation time	
assay	labeling reagent	affinity	antibody	secondary antibody		The name or designation of the secondary antibody	
assay	labeling reagent	affinity	antibody	secondary antibody species		secondary antibody species	
assay	labeling reagent	affinity	antibody	secondary antibody source		secondary antibody source	
assay	labeling reagent	affinity	antibody	secondary antibody lot number		secondary antibody lot number	
assay	labeling reagent	affinity	antibody	secondary antibody working concentration		secondary antibody working concentration	
assay	labeling reagent	affinity	antibody	secondary antibody incubation time		secondary antibody incubation time (in hours)	
assay	labeling reagent	affinity	non-antibody	non-antibody affinity reagent name		Highly reused terms describing the non-antibody affinity reagent stain used in the assay	
assay	labeling reagent	affinity	non-antibody	non-antibody affinity reagent source		Source of the non-antibody affinity reagent	
assay	labeling reagent	affinity	non-antibody	non-antibody affinity reagent concentration		Concentration of the non-antibody affinity reagent used in the assay	
assay	labeling reagent	affinity	non-antibody	non-antibody affinity reagent incubation time		in hours	
assay	target			target protein		Highly reused terms describing the protein labeled in the assay	
assay	target			target organelle	nuclei	Highly reused terms describing the organelle labeled in the assay	
assay	target			target nucleic acid		Highly reused terms describing the nucleic acid labeled in the assay	
assay	target			target carbohydrate		Highly reused terms describing the carbohydrate labeled in the assay	
<NOTE- "instrument" is a root term folder that holds all the variables related to microscopy imaging>	
instrument	optical microscope	benchmarks		spatial benchmark		Highly reused words describing the type of device used for spatial benchmarking	
instrument	optical microscope	benchmarks		intensity benchmark		Highly reused words describing the type of material used to benchmark the lamp intensity	
instrument	optical microscope	benchmarks		resolution benchmark		Highly reused words describing the type of device used to benchmark the microscope resolution	
instrument	optical microscope	benchmarks		flat field benchmark		Highly reused words describing the type of benchmark used to generate the flat field	
instrument	optical microscope			microscope manufacturer	Olympus	Manufacturer name	
instrument	optical microscope			microscope model	IX71	microscope model name	
instrument	optical microscope			microscope orientation	inverted	Highly reused words describing the orientation of the microscope.	
instrument	optical microscope			imaging mode	fluorescence	Highly reused words describing the imaging mode being used to collect the image series	
instrument	optical microscope	light source	excitation	excitation source manufacturer	Olympus	Manufacturer name of the excitation light source	
instrument	optical microscope	light source	excitation	excitation source model	U-LH75XEAPO	Model name of the excitation light source	
instrument	optical microscope	light source	transmission	transmission light type		Highly reused words describing the transmission light source	
instrument	optical microscope	light source	transmission	tranmission light source power		Manufacturers power rating in watts	
instrument	optical microscope	light source	excitation	excitation source type	Xe	Highly reused words describing the excitation light source	
instrument	optical microscope	light source	excitation	excitation source power	75	Manufacturers power rating in watts	
instrument	optical microscope	light source	excitation	radiant power		The measured total power being emitted from the objective with filters in place (in watts)	
instrument	optical microscope	detector	ccd	ccd manufacturer	Roper Scientific	Manufacturer name	
instrument	optical microscope	detector	ccd	ccd model	CoolSnap HQ	Model name	
instrument	optical microscope	detector	ccd	ccd gain	1	CCD camera gain value	
instrument	optical microscope	detector	ccd	ccd offset		CCD camera offset value	
instrument	optical microscope	detector	ccd	ccd exposure time	2	in milliseconds	
instrument	optical microscope	detector	ccd	ccd binning	2x2	The number of pixels (x and y direction) that are binned during the image series collection	
instrument	optical microscope	lens	objective	objective lens manufacturer	Olympus	Manufacturer name	
instrument	optical microscope	lens	objective	objective lens model	UPLANAPO	Model name	
instrument	optical microscope	lens	objective	numerical aperture	0.4	Numerical aperture of the 	
instrument	optical microscope	lens	objective	magnification	10	Fold-magnification of the objective	
instrument	optical microscope	filter	excitation	excitation filter manufacturer	Chroma Technology Corporation	Manufacturer name	
instrument	optical microscope	filter	excitation	excitation filter model 	D360_40x	Model designation	
instrument	optical microscope	filter	excitation	excitation filter lot		as provided by manufacturer	
instrument	optical microscope	filter	excitation	excitation filter type	notch	Highly reused words that describe the type of emission filter (bandpass, notch, high pass)	
instrument	optical microscope	filter	excitation	excitation center wavelength	360	in nanometers	
instrument	optical microscope	filter	excitation	excitation spectral width at half maximum	40	in nanometers	
instrument	optical microscope	filter	excitation	excitation filter custom modification		Highly reused to describe the custom filter modification	
instrument	optical microscope	filter	dichroic	dichroic filter manufacturer	Chroma Technology Corporation	Manufacturer name	
instrument	optical microscope	filter	dichroic	dichroic filter model	bs51019+400dclp	Model designation	
instrument	optical microscope	filter	dichroic	dichroic filter lot		as provided by manufacturer	
instrument	optical microscope	filter	dichroic	dichroic center wavelength	360+470+525+790	in nanometers.  A rule could be developed for describing multipass dichroic filters.	
instrument	optical microscope	filter	dichroic	dichroic custom modification	400 dclp	Model designation	
instrument	optical microscope	filter	emission	emission filter manufacturer	Chroma Technology Corporation	Manufacturer name	
instrument	optical microscope	filter	emission	emission filter model	D460_40x	Model designation	
instrument	optical microscope	filter	emission	emission filter lot		as provided by manufacturer	
instrument	optical microscope	filter	emission	emission filter type 	notch	Highly reused words that describe the type of emission filter (bandpass, notch, high pass)	
instrument	optical microscope	filter	emission	emission center wavelength	460	in nanometers	
instrument	optical microscope	filter	emission	emission spectral width at half maximum	40	in nanometers	
instrument	optical microscope	filter	emission	emission filter custom modification		Highly reused and short words to describe the custom filter modification 	
