# Supplementary material for: New concepts for building vocabulary for cell image ontologies
Source: BMC Bioinformatics. 2011 Dec 21;12:487. doi: 10.1186/1471-2105-12-487 (PMC3293096; doi:10.1186/1471-2105-12-487)
Supplement: Additional File 2 — Adding new metadata terms to the existing ontology. New terms are added to describe time lapse data. The new lines of terms can be added to the end of the existing metadata list or data table shown in Additional File 1. The new terms (which are highlighted) are associated with appropriate existing root terms as shown. When the data tree is generated from the data table, terms are grouped into common nodes defined by the root terms. [file 1471-2105-12-487-S2.PPT]

## Slide 1
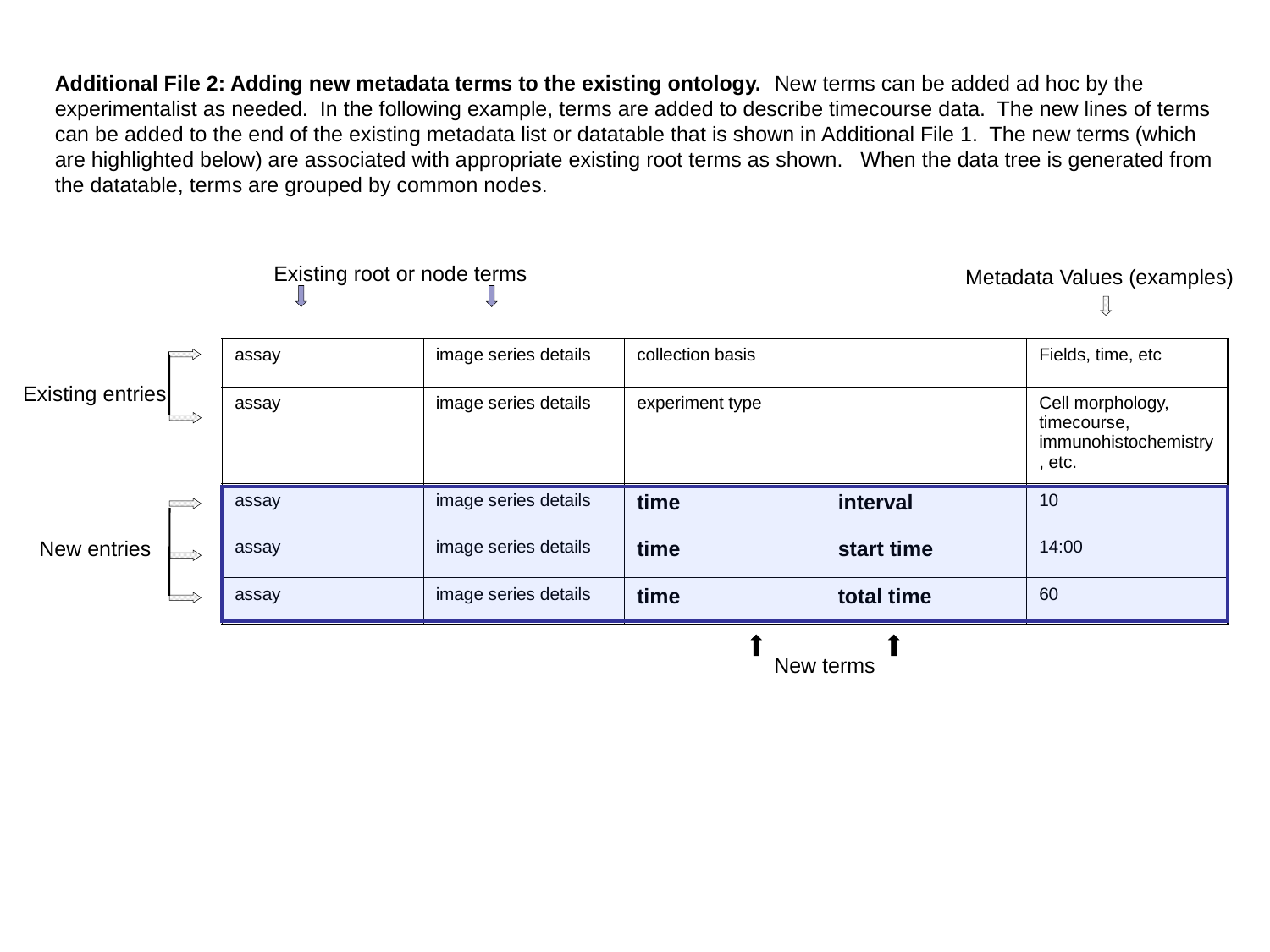

Additional File 2: Adding new metadata terms to the existing ontology. New terms can be added ad hoc by the experimentalist as needed. In the following example, terms are added to describe timecourse data. The new lines of terms can be added to the end of the existing metadata list or datatable that is shown in Additional File 1. The new terms (which are highlighted below) are associated with appropriate existing root terms as shown. When the data tree is generated from the datatable, terms are grouped by common nodes.
Existing root or node terms
Metadata Values (examples)
| assay | image series details | collection basis | | Fields, time, etc |
| --- | --- | --- | --- | --- |
| assay | image series details | experiment type | | Cell morphology, timecourse, immunohistochemistry, etc. |
| assay | image series details | time | interval | 10 |
| assay | image series details | time | start time | 14:00 |
| assay | image series details | time | total time | 60 |
Existing entries
New entries
New terms
