# Supplementary material for: New concepts for building vocabulary for cell image ontologies
Source: BMC Bioinformatics. 2011 Dec 21;12:487. doi: 10.1186/1471-2105-12-487 (PMC3293096; doi:10.1186/1471-2105-12-487)
Supplement: Additional File 3 — Data Tables used for Linkage between Metadata and Image and Protocol Files. Data tables showing linkage between metadata terms and unique filenames for the image and protocol data. Three tables are generated during the upload of the metadata template file, the image file series and the protocol files. The file series name connects the tables. [file 1471-2105-12-487-S3.PPT]

## Slide 1
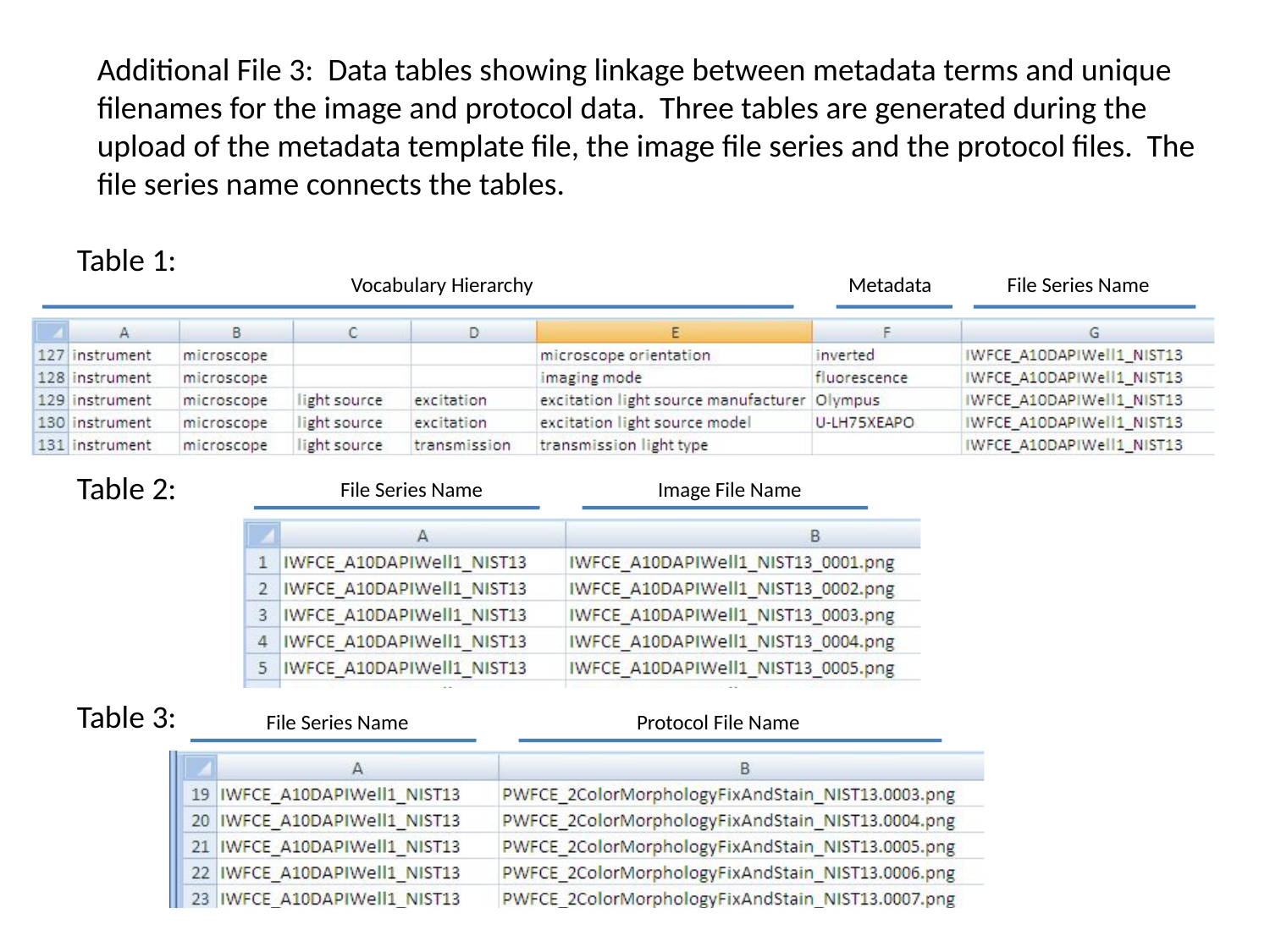

Additional File 3: Data tables showing linkage between metadata terms and unique filenames for the image and protocol data. Three tables are generated during the upload of the metadata template file, the image file series and the protocol files. The file series name connects the tables.
Table 1:
Table 2:
Table 3:
Vocabulary Hierarchy
Metadata
File Series Name
File Series Name
Image File Name
File Series Name
Protocol File Name
