# Supplementary material for: New concepts for building vocabulary for cell image ontologies
Source: BMC Bioinformatics. 2011 Dec 21;12:487. doi: 10.1186/1471-2105-12-487 (PMC3293096; doi:10.1186/1471-2105-12-487)
Supplement: Additional File 4 — Description of the Prototype Database Screens. The url for the database is http://sbd.nist.gov/image/cell_image.html. This file contains information about navigating the web page. [file 1471-2105-12-487-S4.DOC]

**Additional File 3: Description of the Prototype Database Screens.**

Below is a description of the screens for navigating the prototype Cell Image Database. The user can expand folders to view metadata terms of high granularity that including identification of benchmark materials, and detailed information about the history of the cell culture. Users can create queries by choosing terms from the expandable folders, can visualize all metadata associated with particular images, and can do logical operations to compare metadata from different experiments. The data in the prototype database (http://sbd.nist.gov/image/cell_image.html) are NIST Standard Reference Data #171.

**The number designations in Figure 4 are described below:**

**1. Metadata Selection Panel:** All metadata terms and values for the image sets stored in the database can be reached using this tree structure. The top-level folders are identified with the root terms ‘*assay*’, ‘*study*’, ‘*instrument*’ and ‘*cell*’. Each folder expands to a viewable and selectable list of terms of increasingly greater granularity that lead to metadata values. Only metadata terms that have values assigned to the currently selected database or dataset are viewable by the user.

**2. Image Selection Panel:** When a metadata value is selected or a metadata value query is processed, the resulting images are shown here. If only one image series is returned, then each image in the image series is displayed. If more than one image series is returned, then only the first image of each series is shown. All metadata values for that image series are shown in the Metadata Viewing Panel when an image is clicked on. Image series can be compared by clicking on multiple images and performing a query.

**3. Protocol Viewer Panel:** Free-form text protocols that are associated with the image series are shown in the protocol viewing panel. If a single image series has been selected, then all the protocols associated with that image series are shown. If multiple image series are being compared, then protocols from all image series are shown.

**4. Query and Download Panel:** The orange metadata and image series query panel is used to develop queries on metadata terms and to compare image series. This panel is also used to select image series, protocols and associated metadata for downloading.

**5. Keyword Search Panel:** The keyword search panel is a free word “Google-like” search tool to locate metadata terms. If the submitted keyword is a term in the vocabulary, then the results will be displayed in the Keyword Locator Panel.

**6. Keyword Locator Panel:** This panel shows the results of a keyword search. If the submitted keyword is identified in the metadata or hierarchy terms, then the location of the term in the metadata tree is displayed. The level0, level1, level2, etc represent the hierarchy folders shown in the Metadata Selection Panel.

**7. Metadata Viewer Panel:** If a single image series is the result of a query or has been selected in the Image Selection Panel, then this panel shows all the metadata terms and values for the corresponding image series. If multiple image series have been selected, then this panel shows a comparison of the metadata terms between each pair of image series. Only metadata terms and values that are different between any two image series are shown. The image ID information in the table is used to identify which two image series are being compared.

Additional information about the use of the webpage can be found in the Help File which is accessible from the database webpage (http://sbd.nist.gov/image/cell_image.html).
